# Overexpression of COL3A1 confers a poor prognosis in human bladder cancer identified by co-expression analysis

**DOI:** 10.18632/oncotarget.19733

**Published:** 2017-07-28

**Authors:** Lushun Yuan, Bo Shu, Liang Chen, Kaiyu Qian, Yongzhi Wang, Guofeng Qian, Yuan Zhu, Xinyue Cao, Conghua Xie, Yu Xiao, Xinghuan Wang

**Affiliations:** ^1^ Department of Urology, Zhongnan Hospital of Wuhan University, Wuhan, China; ^2^ Department of Urology, The Central Hospital of Wuhan, Tongji Medical College, Huazhong University of Science and Technology, Wuhan, China; ^3^ Department of Urology, The Fifth Hospital of Wuhan, Wuhan, China; ^4^ Department of Endocrinology, The First Affiliated Hospital of Zhejiang University, Hangzhou, China; ^5^ Laboratory of Precision Medicine, Zhongnan Hospital of Wuhan University, Wuhan, China; ^6^ Department of Biological Repositories, Zhongnan Hospital of Wuhan University, Wuhan, China; ^7^ Department of Radiation and Medical Oncology, Zhongnan Hospital of Wuhan University, Wuhan, China

**Keywords:** COL3A1, bladder cancer, co-expression analysis, MAPK, clinicopathology

## Abstract

Human bladder cancer (BCa) is one of the worldwide cancers in men and women populations, with the etiology and mechanism unknown. In our study, we constructed a weighted gene co-expression network to identify gene modules associated with the progression of BCa (n = 93). In the significant module (R^2^ = 0.48), a total of 103 network hub genes were identified, and 4 of them were hub nodes in the protein-protein interaction network as well. In validation, *COL3A1* showed a higher correlation with the disease progression than any other hub genes in hub module in the test set (p < 0.001). Functional and pathway enrichment analysis demonstrated that *COL3A1* is overrepresented in pathway of focal adhesion, which associated with tumor progression and might cause metastasis. Gene set enrichment analysis (GSEA) also demonstrated that the gene set of “MAPK signaling pathway” and focal adhesion related pathways were enriched in BCa samples with *COL3A1* highly expressed (FDR < 0.05). Considering the clinicopathological parameters, highly-expressed *COL3A1* was closely correlated with local recurrence and BCa stage. Survival analysis revealed that BCa patients with higher expression of *COL3A1* had a significantly shorter overall survival time and disease free survival time.In conclusion, based on the co-expression analysis, *COL3A1* was identified in the association with progression and prognosis of BCa, which might refer a poor prognosisprobably by regulating MAPK signaling pathway.

## INTRODUCTION

Bladder cancer is one of the worldwide cancers in men and women populations. The risk factors are different, including tobacco use, Schistosoma infection, chemical exposure, diet and lifestyle trends, atmospheric pollution and genetic susceptibilities, so the incidence of bladder cancer has a considerable global variation [1, 2]. In Western countries, the total incidence of urothelial carcinoma accounts for 90% [3]. In China, bladder cancer is reported to be the most common genitourinary malignancy, and its incidence has increased rapidly in the last few decades [4]. Although patients underwent complex surgery and various adjuvant treatments, 5-year survival rates of BCa are only 60% [5]. In addition, it has also been reported that 30 - 70% of those tumors have a chance of recurrence, and up to 30% of the population quickly progress to muscle-invasive disease [6]. At initial diagnosis, bladder cancer could be characterized by 2 histological subtypes: non-muscle-invasive bladder cancer (NMIBC) and muscle-invasive bladder cancer (MIBC) [7]. 10% to 30% of patients with NMIBC recur and progress to MIBC, which is responsible for most bladder cancer-specific deaths [8]. As MIBC frequently causes distant metastases, it is urgent to understand the mechanisms that promote cancer progression and find novel molecular markers for the early diagnosis and prognosis.

With the development of high-throughput microarray technology, gene expression profiles have been used to identify genes associated with progression of bladder cancer. Nowadays, most studies just concentrated on the screening differentially expressed genes and not attached enough attention to the high degree of interconnection between genes, where genes with similar expression patterns may be functionally related. The algorithm, weighted gene co-expression network analysis (WGCNA) can construct free-scale gene co-expression networks to explore the relationships between different gene sets or between gene sets and clinical features. Thus, we attempt to construct a co-expression network of relationships between genes through a systematic biology method based on a weighted genome expression network [9] and to identify network-centric genes associated with clinical features of bladder cancer.

## RESULTS

### Differentially expressed genes (DEGs) screening

**Figure 1:**
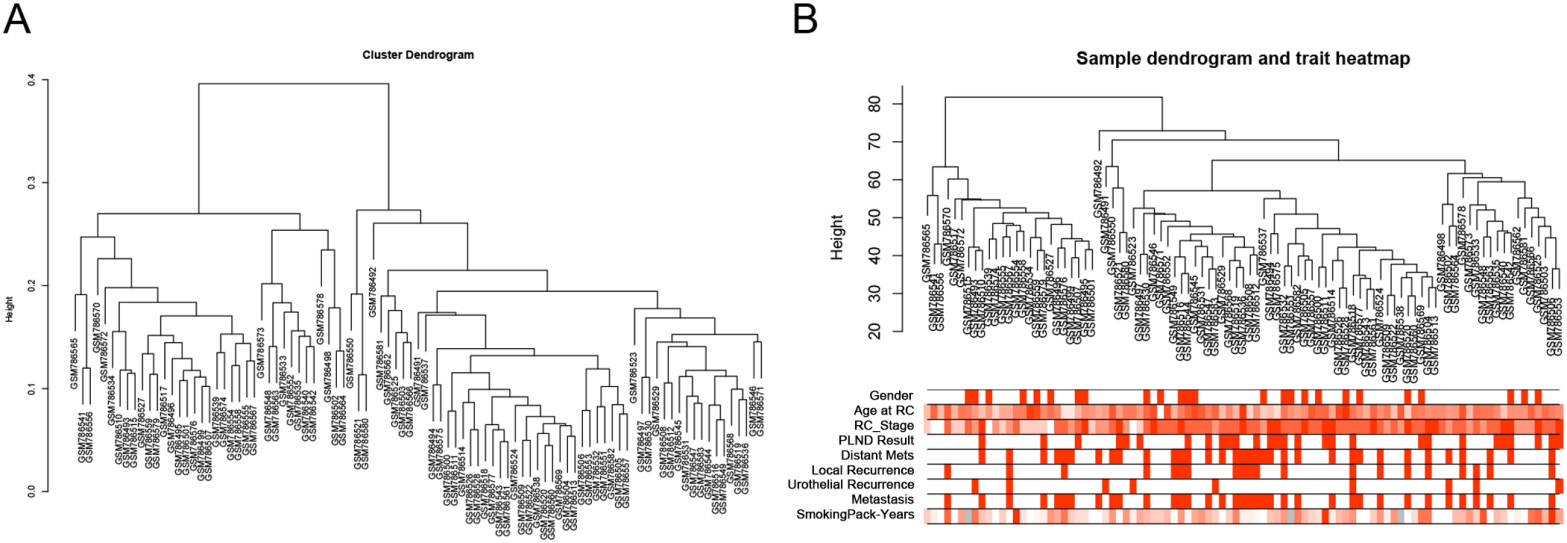
Samples clustering to detect outliers (GSE31684) **(A)** Cluster dendrogram. **(B)** Sample dendrogram and trait indicator. The clustering was based on the expression data of differentially expressed genes between low grade and high grade tumor samples in BCa. The color intensity was proportional to age at RC, RC stage and smoking pack-years. (RC: radical cystectomy).

After data preprocessing and quality assessment, the expression matrices were obtained from the 93 samples in GSE31684 (Figure 1). Under the threshold of FDR < 0.05 and |log_2_FC| > 0.263, a total of 874 DEGs (454 up-regulated or 420 down-regulated) were selected for subsequent analysis.

### Weighted co-expression network construction and key modules identification

**Figure 2:**
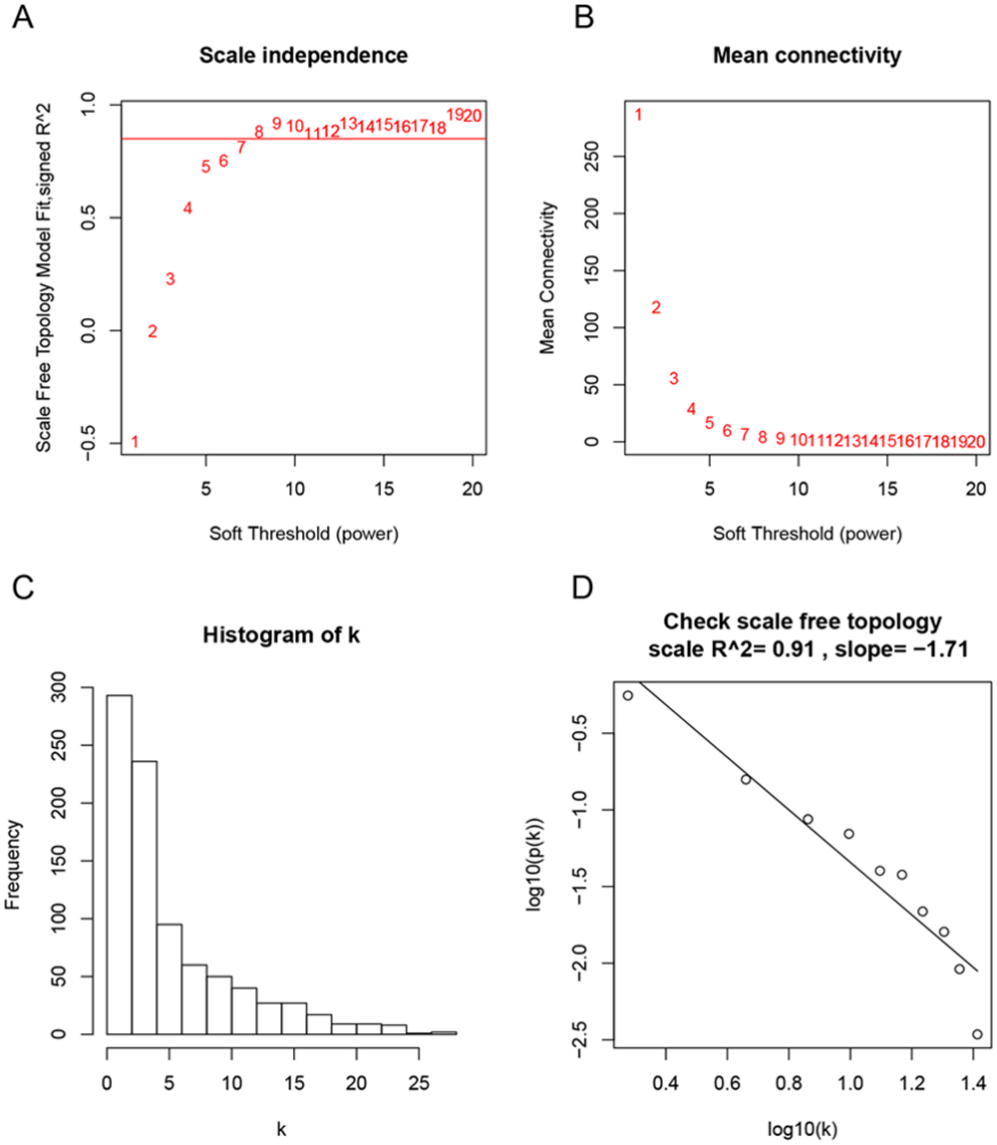
Determination of soft-thresholding power in the weighted gene co-expression network analysis (WGCNA) **(A)** Analysis of the scale-free fit index for various soft-thresholding powers (β). **(B)** Analysis of the mean connectivity for various soft-thresholding powers. **(C)** Histogram of connectivity distribution when β = 8. **(D)** Checking the scale free topology when β = 8.

**Figure 3:**
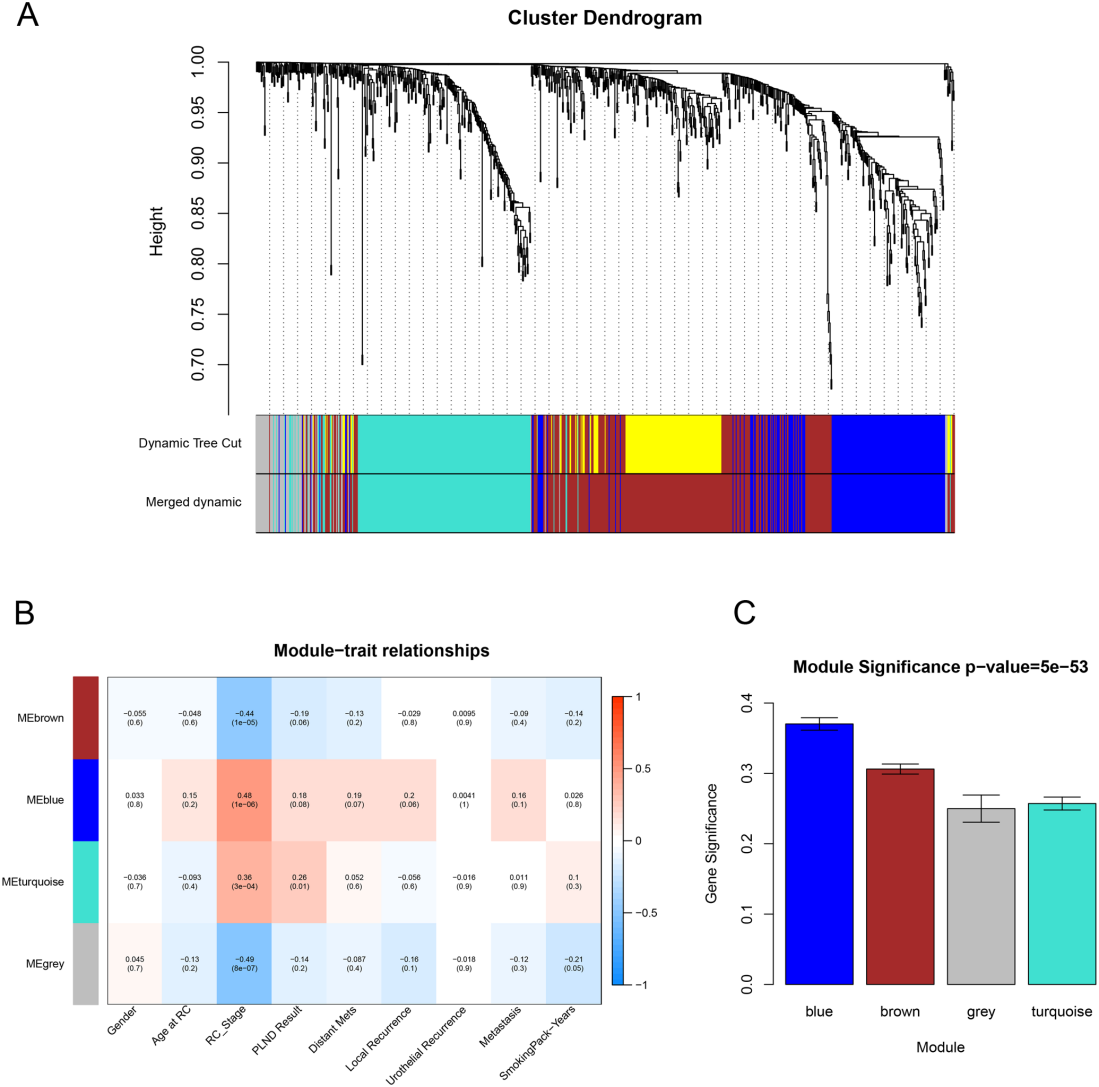
Identification of modules associated with clinical information **(A)** Dendrogram of all differentially expressed genes clustered based on a dissimilarity measure (1-TOM). **(B)** Heatmap of the correlation between module eigengenes and different clinical information of BCa (Gender, Age at RC, RC_Stage, PLIND Results, Distant Mets, Local Recurrence, Urothelial Recurrence, Metastasis and SmokingPack-Years). **(C)** Distribution of average gene significance and errors in the modules associated with the progression of BCa.

We used “WGCNA” package (Figure 2) in R to put the DEGs with similar expression patterns into modules by average linkage clustering, 5 modules were identified at first, choosing a cutline of 0.1, a total of 4 module were finally identified (Figure 3A). Two methods were used to test the relevance between each module and the clinical information we interested. Here, we chose stage of tissue after radical cystectomy (RC_Stage) as our interested clinical information. We found that the module eigengene (ME) in the blue module showed a higher correlation with RC_Stage than other modules (Figure 3B). As modules with greater module significance (MS) were considered to have more connection with our concerned clinical information, we found that the MS of blue module was also higher than those of any other MS (Figure 3C). Based on the two methods, we identified the blue module was the module most relevant to the disease progression of BCa.

### Hub gene identification

**Figure 4:**
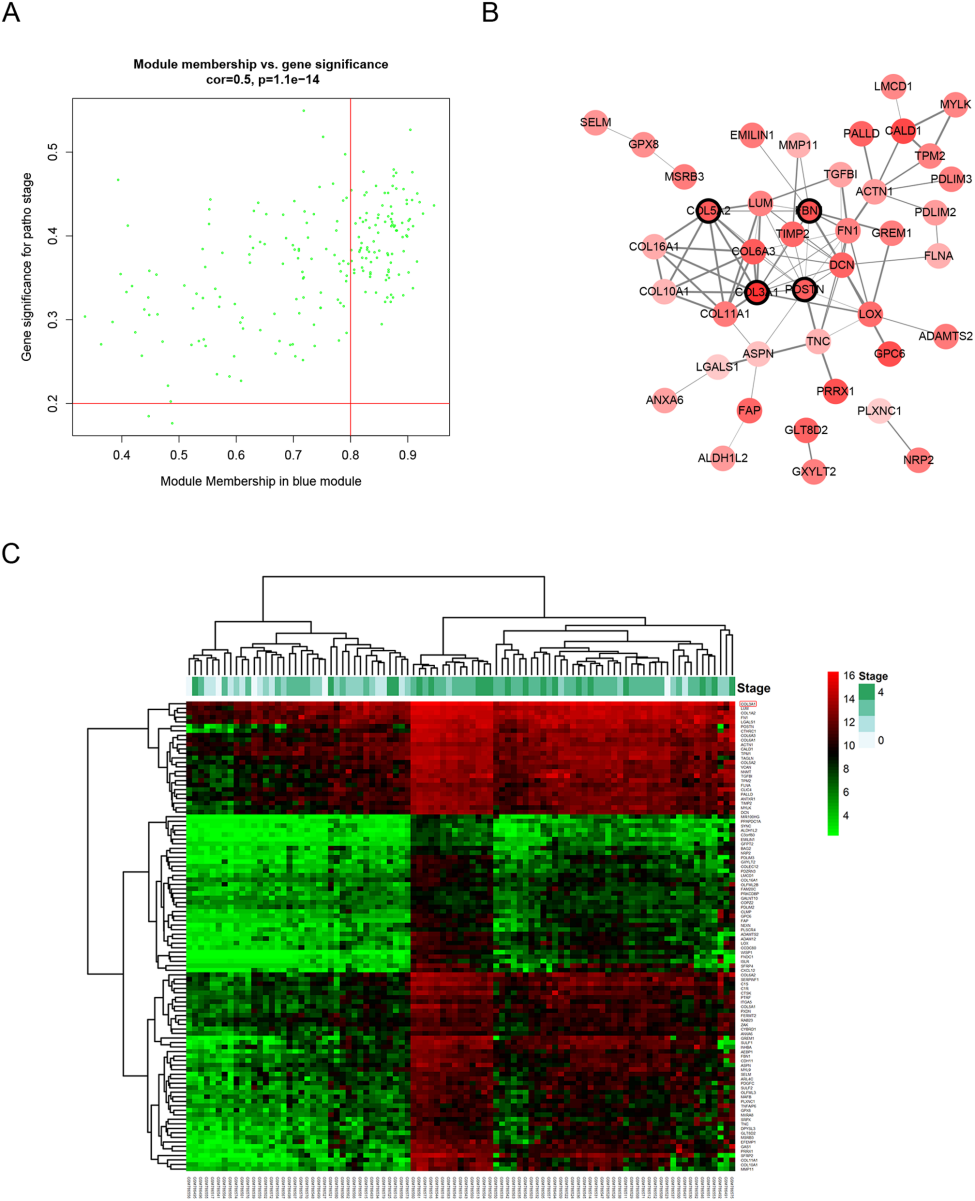
Hub genes detection and protein-protein network (PPI) **(A)** Scatter plot of module eigengenes in blue module. **(B)** Protein-protein interaction network of genes in the blue module. The color intensity in each node was proportional to the degree of connectivity in the weighted gene co-expression network (positive correlation in red and negative correlation in green, in our PPI network, there’s no negative correlation node). The nodes with bold circle represented network hub genes identified by WGCNA. The edge width was proportional to the score of protein-protein interaction based on the STRING database. **(C)** Heatmap of the expression of hub genes in different stages of BCa.

Defined by module connectivity, measured by absolute value of the Pearson’s correlation (cor.geneModuleMembership > 0.8) and clinical trait relationship, measured by absolute value of the Pearson’s correlation (cor.geneTraitSignificance > 0.2), 103 genes with the high connectivity in blue module were taken as hub genes (Figure 4A and 4C). Moreover, we also constructed a network of protein-protein interaction (PPI) for all hub genes in blue module by Cytoscape according to the STRING database, and genes connected with more than 4 nodes were identified as hub nodes in the PPI network (*COL3A1, COL5A2, FBN1* and *POSTN*, Figure 4B).

### Hub gene validation

**Figure 5:**
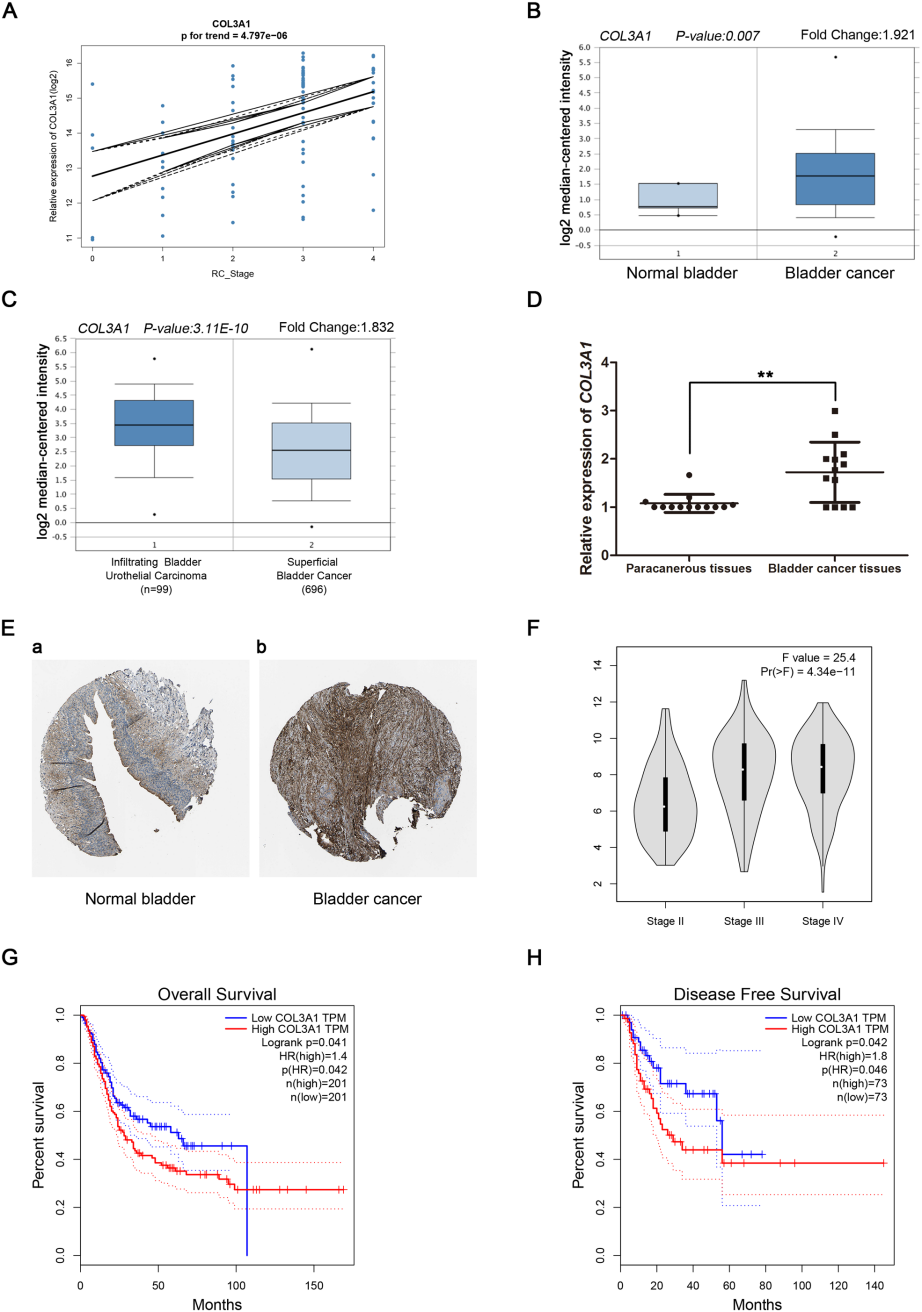
Validation of *COL3A1* **(A)** *COL3A1* expression was correlated with the disease progression of BCa (GSE13507). **(B)** and **(C)** Oncomine database indicated that *COL3A1* was up-regulated in BCa, compared with normal bladder, as well as up-regulated in infiltrating bladder urothelial carcinoma, compared with superficial bladder cancer. **(D)** *COL3A1* mRNA was validated using 13 BCa tissues and matched paracancerous tissues by qRT-PCR (p < 0.01). **(E)** The human protein atlas database indicated the elevated expression of *COL3A1* in BCa tissue compared with normal bladder tissue on translational level. (The normal urinary bladder tissue was from a female, aged 79 (Patient id: 3265; staining: low; intensity: moderate; quantity: < 25%; location: cytoplasmic/membranous) and the bladder cancer tissue was from a female, aged 56 (Patient ID: 3077; staining: medium; intensity: moderate; quantity: 75% - 25%; location: cytoplasmic/membranous) **(F)** GEPIA database indicated that *COL3A1* had a strong correlation with the progression of BCa based on TCGA data. **(G-H)**Kaplan-Meier survival curve obtained GEPIA database revealed that BCa patients with higher expression of *COL3A1* had a significantly shorter (G) overall survival time and (H) disease free survival time.

Linear regression analyses were conducted to validate hub genes in the test set. Comparing with the correlation of *COL3A1* had a higher correlation than other genes (p < 0.001, Figure 5A). Therefore, *COL3A1* was chosen as the candidate gene for further validation. Based on the Oncomine database, we could found that the expression of *COL3A1* was not only highly-expressed in normal bladder, but also had a strong correlation with progression of BCa (Figure 5B and Figure 5C). More convincingly, the result of real-time PCR using 13 BCa tissues and matched paracancerous tissues exhibited a significant up-regulation in BCa tissues than paracancerous tissues (p < 0.01, Figure 5D). Interestingly, immunohistochemistry staining obtained from The Human Protein Atlas database, revealed strong increase of *COL3A1* protein in BCa tissues, compared with normal bladder, and we could also find a trend of increasing intensity of *COL3A1* with the progression of BCa (Figure 5E). Meanwhile, according to the GEPIA database, we could find a significant difference between different stages of BCa (Figure 5F). In addition, we discovered that patients with higher expression of *COL3A1* had a significantly shorter overall survival time and disease free survival time (Figure 5G and 5H). Interestingly, using supplemary data in test set, we also performed the survival analysis, which revealed the same result (Supplementary Figure 1A-1B). Comparing different clinicopathological parameters, we found that *COL3A1* was significantly altered in different stages of BCa; higher expression of *COL3A1* could cause more local recurrences; meanwhile, higher expression of *COL3A1* could result in shorter survival time and recurrence free survival time (Table 1). The ROC curves of local recurrence (AUC = 0.666, p = 0.023), recurrence (AUC = 0.626, p = 0.040) and stage (AUC = 0.807, p = 0.001), indicating the clinical value of *COL3A1* expression and BCa progression and recurrence (Supplementary Figure 1C-1E).

**Table 1:** Correlations between COL3A1 expression and clinicopathological parameters in bladder cancer

Parameter	Category	Bladder Cancer		P Value
		Expression of COL3A1		
		Low	High	
Gender	male	34	34	1
	female	12	12	
Age at RC	<65	14	13	0.819
	≥65	32	33	
Stage (T)	Ta	4	1	0.001*
	T1	10	0	
	T2-T4	32	45	
Histological Grade	Low Grade	5	1	0.091
	High Grade	41	45	
PLND Result	Positive	12	16	0.78
	Negative	34	30	
SmokingPack-Years		47.21±31.95	41.33±24.95	0.403
Recurrence/DOD	yes	15	24	0.058
	no	31	22	
Survival Months		59.87±48.63	36.05±36.27	0.010*
Recurrence Free Survival Months (Distant and Local)		58.42±49.48	28.36±33.98	0.001*
Distant Mets	yes	13	21	0.084
	no	33	25	
Metastasis	yes	14	22	0.087
	no	32	24	
Local Recurrence	yes	6	14	0.043*
	no	40	32	
Urothelial Recurrence	yes	5	4	0.726
	no	41	42	
PreRC_Chemo	yes	3	0	0.078
	no	43	46	
Post RC_Chemo	yes	13	22	0.053
	no	33	24	
Last known status	DOD	15	23	0.137
	NED	18	10	
	DOC	13	13	

### Functional and pathway enrichment analysis

**Figure 6:**
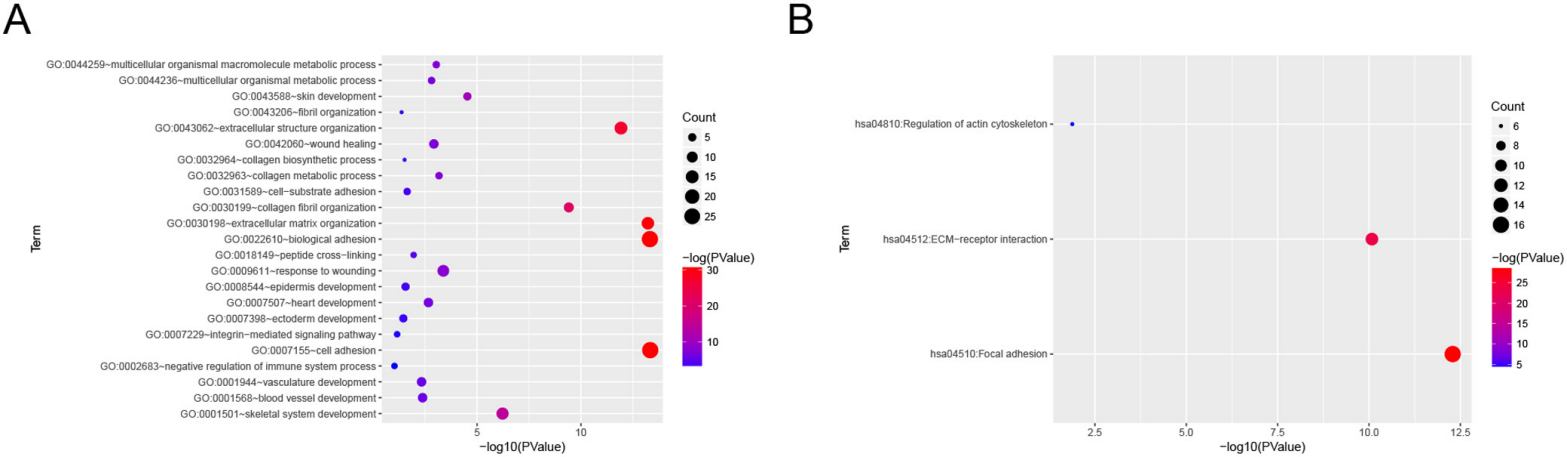
Bioinformatics analysis of Differentially expressed genes (DEGs) **(A)** GO analysis and **(B)** KEGG pathway enrichment of *COL3A1*.

To obtain further insight into the function of DEGs in hub module, they were uploaded to the Database for Annotation, Visualization and Integrate Discovery (DAVID) database (*http://david.abcc.ncifcrf.gov/*) [10]. GO analysis results showed that *COL3A1* were enriched in 23 biological processes (BP, Supplementary Table 1), and among those, we found that *COL3A1* was significantly enriched in extracellular structure organization, extracellular matrix organization, biological adhesion and cell adhesion. Moreover, *COL3A1* was overrepresented in 3 KEGG pathways (Supplementary Table 2), including regulation of actin cytoskeleton, ECM-receptor interaction and focal adhesion (Figure 6).

### Gene set enrichment analysis (GSEA)

**Figure 7:**
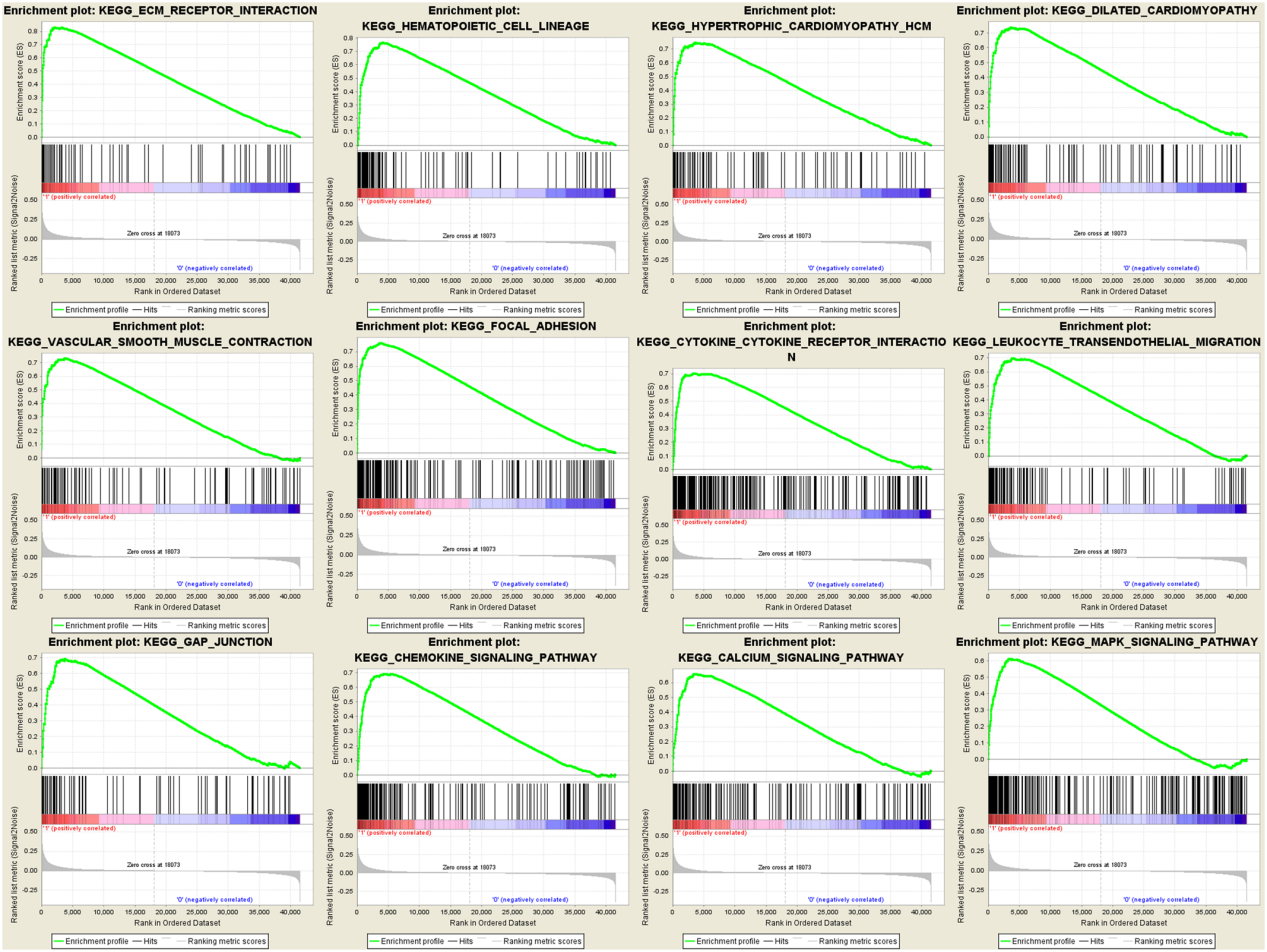
Gene set enrichment analysis (GSEA) analysis for gene sets related with *COL3A1* expression The gene sets of “ECM_RECEPTOR_INTERACTION”, “HEMATOPOIETIC_CELL_LINEAGE”, “FOCAL_ADHESION”, “HYPERTROPHIC_CARDIOMYOPATHY_HCM”, “DILATED_CARDIOMYOPATHY”, “VASCULAR_SMOOTH_MUSCLE_CONTRACTION”, “CYTOKINE_CYTOKINE_RECEPTOR_INTERACTION”, “LEUKOCYTE_TRANSENDOTHELIAL_MIGRATION”, “GAP_JUNCTION”, “CHEMOKINE_SIGNALING_PATHWAY”, “CALCIUM_SIGNALING_PATHWAY” and “MAPK_SIGNALING_PATHWAY” were enriched in BCa samples with *COL3A1* highly expressed (GSE13507).

To identify potential function of the hub genes, GSEA was conducted respectively to search Kyoto Encyclopedia of Genes and Genomes (KEGG) pathways enriched in the samples with the *COL3A1* highly expressed. FDR ≤ 0.05 and gene limits ≥ 80, a total of 12 functional gene sets were enriched in the samples with high expression levels of *COL3A1*. *COL3A1* was enriched “ECM_RECEPTOR_INTERACTION”, “HEMATOPOIETIC_CELL_LINEAGE”, “FOCAL_ADHESION”, “HYPERTROPHIC_CARDIOMYOPATHY_HCM”, “DILATED_CARDIOMYOPATHY”, “VASCULAR_SMOOTH_MUSCLE_CONTRACTION”, “CYTOKINE_CYTOKINE_RECEPTOR_INTERACTION”, “LEUKOCYTE_TRANSENDOTHELIAL_MIGRATION”, “GAP_JUNCTION”, “CHEMOKINE_SIGNALING_PATHWAY”, “CALCIUM_SIGNALING_PATHWAY” and “MAPK_SIGNALING_PATHWAY” (Figure 7). Interestingly, we found that *COL3A1* had a strong correlation with the key molecules of MAPK pathway by regression analysis (Supplementary Figure 2).

## DISCUSSION

Bladder cancer is the most frequent malignant tumor in urinary tract. BCa is still easy to recurre after combined therapy and eventually progressions into infiltrating BCa with poorer prognosis and higher mortality [11]. Currently, radical cystoprostatectomy is the most effective treatment for infiltrating bladder urothelial carcinoma but this therapy will arise a lot of adverse outcomes and the quality of patient's life will be greatly reduced [12]. Tumor stage is an important clinical index for cancer and it is closely related to tumor malignance and metastasis. There is currently no tumor marker that can accurately predict the progression of BCa. Therefore, effective biomarkers for BCa stage prognosis are urgently needed.

In this study, WGCNA was performed to identify gene co-expression modules related with the progression of BCa. The blue module was identified, and 103 hub genes were derived from the module. Furthermore, relating the results of PPI network, *COL3A1, COL5A2, FBN1* and *POSTN* were hub nodes in the both co-expression module and PPI network, indicating that those hub genes had high connection with clinical trait as well as vital biological processes. In validation, *COL3A1* was highly correlated with the clinical trait estimated by log rank test (Figure 5A).

Collagen Type III Alpha 1 Chain (*COL3A1*), encodes the pro-alpha1 chains of type III collagen, a fibrillar collagen that is found in extensible connective tissues such as skin, lung, uterus, intestine and the vascular system, frequently in association with type I collagen [13]. The family of collagen was also reported to play a critical role in tumor progression, migration and proliferation [14]. Mentioning the role of *COL3A1* in tumorgenesis, Yoshida *et al.* reported that *COL3A1* as the novel translocation partner gene of *PLAG1* could differentiate lipomatous tumors by influencing *PLAG1* rearrangement and overexpression [15]. Qiu *et al.* reported that miR-29a/b promotes cell migration and invasion in nasopharyngeal carcinoma progression by regulating *SPARC* and *COL3A1* gene expression [16]. Moreover, Su *et al.* discovered that let-7d suppresses growth, metastasis, and tumor macrophage infiltration in renal cell carcinoma by targeting *COL3A1* and *CCL7* [17]. Interestingly, *COL3A1* was reported to be one of etiologically linked genes in isolated vasculopathies such as aortic dissected aneurysm or CAD [18].

As an oncogene, *COL3A1* was highly expressed in bladder cancer tissues, compared with normal bladder. To obtain further insight of translational level of the expression of *COL3A1*, we observed the immunohistochemistry staining of *COL3A1* in both normal bladder and bladder cancer in the Human Protein Atlas database and discovered that compared with normal bladder tissue, the expression of *COL3A1* was significantly up-regulated in bladder cancer tissue. To verify the results of the expression of *COL3A1* in transcriptional level, we used 13 pairs of BCa tissues and paracanerous tissues to perform real-time PCR, and the results showed that the expression of BCa tissues was significantly up-regulated comparing with the paracancerous tissues (p < 0.001). Moreover, we found a significant difference of expression of *COL3A1* between infiltrating bladder urothelial carcinoma and superficial bladder cancer, indicating that *COL3A1* maybe correlated with the invasive bladder cancer and non-invasive bladder cancer. Based on the GEPIA database, we could also found that the expression of *COL3A1* increased with the progression of BCa. Combined with the expression of NMIBC and MIBC, we supposed that COL3A1 played an important role in the progression of BCa and may be a candidate biomarker to distinguish NMIBC and MIBC. As to the prognostic, we could observe that higher expression of *COL3A1* cause lower survival rate and shorter overall survival time and disease free survival time. The same results were obtained from the test set and TCGA data.

Considering the functional and pathway enrichment analysis, *COL3A1* was overrepresented in cell adhesion, biological adhesion and focal adhesion. Meanwhile, GSEA analysis demonstrated that genes associated with focal adhesion and MAPK signaling pathway were enriched in bladder cancer samples with *COL3A1* highly expressed, suggesting that *COL3A1* might affect cell migration or invasion through MAPK signaling pathway. Many studies had reported that the family of collagen plays a vital role in carcinogenesis [19–21]. As a result, we thought that *COL3A1* played certain role in the progression of human bladder cancer and influenced the prognosis probably by regulating MAPK signaling pathway [22], which contributed to the poor prognosis of BCa. Interestingly, according to the correlations between *COL3A1* expression and clinicopathological parameters in bladder cancer, we found that highly expressed *COL3A1* could cause the progression of BCa and more local recurrence rate.

In conclusion, our study used weighted gene co-expression analysis to construct a gene co-expression network, identify and validate network hub genes associated with the progression and poor prognosis of BCa. Eventually, *COL3A1* was identified and validated in association with the progression and poor prognosis of BCa probably by regulating MAPK signaling pathway.

## MATERIALS AND METHODS

### Data collection

Raw expression data of bladder cancer were downloaded from Gene Expression Omnibus (GEO) database (*http://www.ncbi.nlm.nih.gov/geo/*). Dataset GSE31684 [23] performed on Affymetrix Human Genome U133 Plus 2.0 Array (Affymetrix, Santa Clara, CA, USA) was used to construct co-expression networks and identify hub genes in this study. This dataset included microarray data of 93 bladder cancer patients managed by radical cystectomy (including bladder cancer of pathological Ta, T1, T2, T3 and T4) with clinical and prognostic variables. Another dataset of GSE13507 [24] was downloaded to use as a test set to verify our results. This dataset included 58 normal bladder samples, 165 primary bladder cancer samples and 23 recurrent non-muscle invasive tumor tissues.

### Data preprocessing

For the analyses, raw expression data were firstly performed RMA background correction, and their processed signals were log_2_ transformed and normalized by quantile normalization. Then median-polish probesets were summarized by using the “affy” [25] R package. Probes were annotated by the Affymetrix annotation files. Microarray quality was assessed by sample clustering according to the distance between different samples in Pearson’s correlation matrices and average linkage, and no samples were removed from subsequent analysis in GSE31684 (Figure 1A and 1B).

### Identification of differentially expressed genes (DEGs)

The “limma” [26] R package was used to screen the DEGs between low stage bladder cancers (Ta-T1) and high stage bladder cancers (T2-T4) in the expressing data. The SAM (significance analysis of microarrays) with FDR (false discovery rate) < 0.05 and |log_2_fold change (FC)| > 0.263 were selected to perform further analysis.

### Co-expression network construction

First of all, we checked the expression data profile of DEGs if they were the good samples or good genes. Then, “WGCNA” package in R was used to construct co-expression network for the DEGs. At first, the Pearson’s correlation matrices were both performed for all pair-wise genes. Then, a weighted adjacency matrix was constructed using a power function a_mn_ = |c_mn_|^β^ (c_mn_ = Pearson’s correlation between gene m and gene n; a_mn_ = adjacency between gene m and gene n). β was a soft-thresholding parameter that could emphasize strong correlations between genes and penalize weak correlations. Here, the power of β = 8 (scale free R^2^ = 0.91) was selected to ensure a scale-free network (Figure 2). Next, the adjacency was transformed into topological overlap matrix (TOM), which could measure the network connectivity of a gene defined as the sum of its adjacency with all other genes for network generation. To classify genes with similar expression profiles into gene modules, average linkage hierarchical clustering was conducted according to the TOM-based dissimilarity measure with a minimum size (gene group) of 30 for the genes dendrogram. To further analyze the module, we calculated the dissimilarity of module eigengenes, chose a cut line for module dendrogram and merged some modules.

### Identification of clinical significant modules

Two approaches were used to identify modules related with clinical information of BCa. After selecting the interesting clinical information, firstly, Gene significance (GS) was defined as the log10 transformation of the P value (GS = lgP) in the linear regression between gene expression and tumor progression. In addition, module significance (MS) was defined as the average GS for all the genes in a module. In general, the module with the absolute MS ranked first or second among all the selected modules was considered as the one related with clinical trait. Module eigengenes (MEs) were considered as the major component in the principal component analysis for each gene module and the expression patterns of all genes could be summarized into a single characteristic expression profile within a given module. In addition, we calculated the correlation between MEs and clinical trait to identify the relevant module.

### Hub gene analysis and validation

Hub genes, highly interconnected with nodes in a module, have been shown to be functionally significant. In our study, after the interesting module was chosen, hub genes were defined by module connectivity, measured by absolute value of the Pearson’s correlation (cor.geneModuleMembership > 0.8) and clinical trait relationship, measured by absolute value of the Pearson’s correlation (cor.geneTraitSignificance > 0.2). In order to screen a key candidate among the hub genes, a linear regression analysis was performed to calculate the relationship between the hub genes expressions and interesting clinical information and R^2^ was defined as the relationship between them. Moreover, we uploaded all genes in the hub module to the STRING database [27] to construct protein-protein interaction (PPI) to screen hub nodes in PPI network.

To verify our results, we used the clinical information in the microarray data to explore correlations between hub gene expression and clinicopathological parameters in BCa as well as used 2 database: The Human Protein Atlas (*http://www.proteinatlas.org*) and Gene Expression Profiling Interactive Analysis (GEPIA) database [28] (*http://gepia.cancer-pku.cn/*) to perform validation of the expression, immunochemistry staining and prognostic of the candidate hub gene. Meanwhile, we perform ROC curve to present the value of *COL3A1* expression for stage, recurrence and local recurrence to perform survival analysis.

### Functional and pathway enrichment analysis

The DAVID database [10] (*http://david.abcc.ncifcrf.gov/*) is an online program providing a comprehensive set of functional annotation tools for investigators to understand biological meaning behind large list of genes. Hub genes in hub module were uploaded to screen enriched GO terms and KEGG pathway maps [29] by using DAVID database. P < 0.05 was set as the cut-off criterion.

### Gene set enrichment analysis (GSEA)

In the test data set, 165 BCa samples were divided into two groups according to the expression level of hub genes respectively. To identify potential function of the hub gene, GSEA (*http://software.broadinstitute.org/gsea/index.jsp*) [30, 31] was conducted to detect whether a series of priori defined biological processes were enriched in the gene rank derived from DEGs between the two groups. For use with GSEA software, the collection of annotated gene sets of c2.cp.kegg.v6.0.symbols.gmt in Molecular Signatures Database (*MSigDB,* *http://software.broadinstitute.org/gsea/msigdb/index.jsp*) was chosen as the reference gene sets. FDR < 0.05 was chosen as the cut-off criteria. Furthermore, we perform cox regression analysis to validate the correlation between the hub gene and the key molecule in the pathway.

### Preparation for human bladder cancer and paracancerous samples

The BCa and paracancerous tissues samples were collected from patients after surgery at Zhongnan Hospital of Wuhan University. The histology diagnosis was confirmed by two pathologists independently. The BCa and paracancerous tissues were immediately frozen and stored in liquid nitrogen or fixed in 4% PFA after collection. The study using BCa and paracancerous tissue samples for total RNA isolation and qRT-PCR analysis was approved by the Ethics Committee at Zhongnan Hospital of Wuhan University (approval number: 2015029). Informed consent was obtained from all subjects.

### Total RNA isolation

Total RNA from bladder cancer and paracancerous tissues were isolated using RNeasy Mini Kit (Cat. #74101, Qiagen, Germany) according to the manufacturer’s instruction. DNase I digestion (Cat. #79254, Qiagen, Germany) was used in each RNA preparation to remove genomic DNA. After that, total RNA quantity was measured using NanoPhotometer (Cat. #N60, Implen, Germany).

### Quantitative real time PCR (qRT-PCR)

The cDNA was synthesized using 1 μg of total RNA isolated from PCa cells by ReverTra Ace qPCR RT Kit (Toyobo, China) and qRT-PCR was performed using 500 ng cDNA per 20 μl reaction. Each reaction was conducted with iQTM SYBR^®^ Green Supermix (Bio-Rad, China) using 500 ng of cDNA in a final volume of 20 μl. Primers used for *COL3A1*: 5'-GGAGCTGGCTACTTCTCGC-3' (forward), 5'-GGGAACATCCTCCTTCAACAG-3' (reverse), annealing temperature was 60°C. Primers used for *GAPDH* (loading control): 5'-TGCACCACCAACTGCTTAG-3' (forward), 5'-GATGCAGGGATGATGTTC-3' (reverse), annealing temperature was 60°C.

### Statistical analyses

Data were expressed as mean ± SD from three independent experiments. All analyses were performed three times and represent data from three individual experiments. Two-tailed Student’s t-tests and one-way analysis of variance (ANOVA) were used to evaluate the statistical significance of differences of the data. All of the statistical analyses were performed with SPSS 16.0. The statistical significance was set at probability values of p < 0.05.

## SUPPLEMENTARY MATERIALS FIGURES AND TABLES






