# Supplementary material for: Overexpression of COL3A1 confers a poor prognosis in human bladder cancer identified by co-expression analysis
Source: Oncotarget. 2017 Jul 28;8(41):70508–20. doi: 10.18632/oncotarget.19733 (PMC5642573; doi:10.18632/oncotarget.19733)
Supplement: Supplementary file 1 [file oncotarget-08-70508-s001.pdf]

Overexpression of COL3A1 confers a poor prognosis in human bladder cancer identified by co-expression analysis

SUPPLEMENTARY MATERIALS

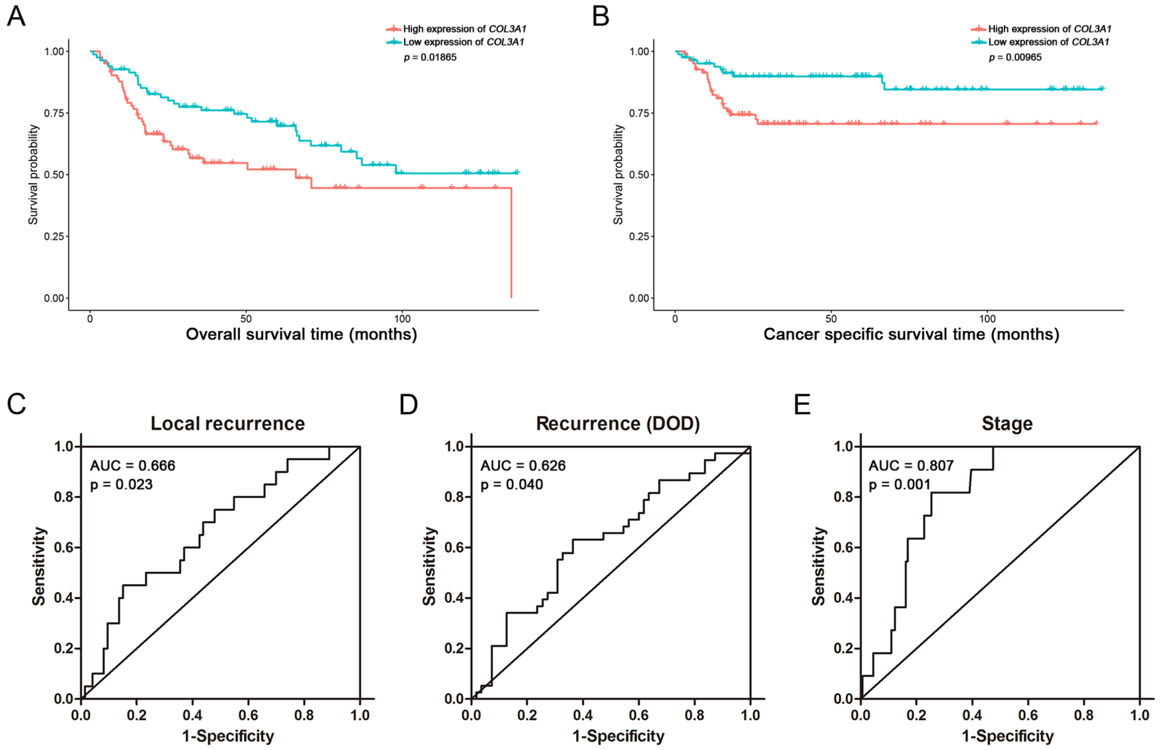

Supplementary Figure 1: Validation of correlation of the correlation between expression of *COL3A1* and clinical features. (A, B) Survival analysis of *COL3A1* using test set (GSE13507). **(C-E)** ROC curves of the expression of *COL3A1* and local recurrence, recurrence and tumor stage.

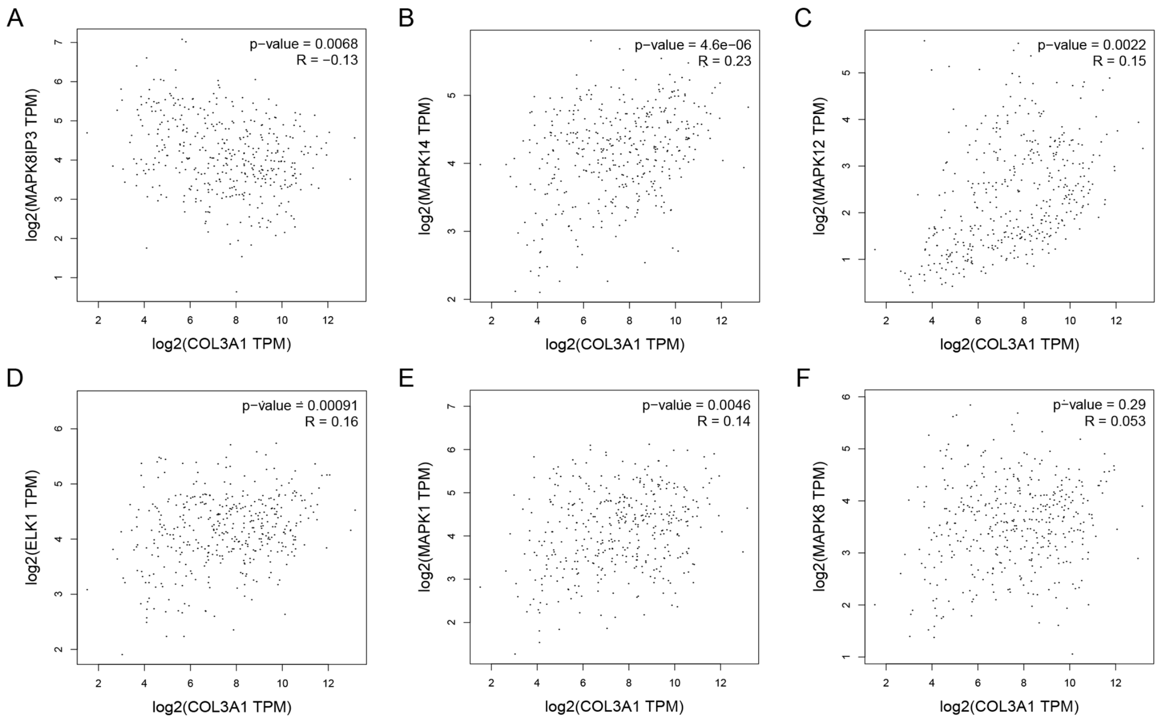

Supplementary Figure 2: Cox regression analysis of the expression of *COL3A1* and key molecules in MAPK pathway. **(A)** *MAPK8IP3*, **(B)** *MAPK14*, **(C)** *MAPK12*, **(D)** *ELK1*, **(E)** *MAPK1* and **(F)** *MAPK8*.

Supplementary Table 1: Enriched biological process of genes in blue module (terms including COL3A1 with highlighted color)

See Supplementary File 1

Supplementary Table 2: Enriched KEGG pathways of genes in blue module

See Supplementary File 2
